# Eye-Movements in a Text Reading Task: A Comparison of Preterm Children, Children with Dyslexia and Typical Readers

**DOI:** 10.3390/brainsci13030425

**Published:** 2023-02-28

**Authors:** Paola Bonifacci, Valentina Tobia, Alessandra Sansavini, Annalisa Guarini

**Affiliations:** 1 Department of Psychology “Renzo Canestrari”, University of Bologna, 40127 Bologna, Italy; 2 Faculty of Psychology, Vita-Salute San Raffaele University, 20132 Milan, Italy

**Keywords:** preterm birth, dyslexia, eye-movements, reading, primary schools

## Abstract

Preterm birth is associated with weaknesses in reading skills that are usually less severe than those of children with dyslexia. To understand the characteristics of reading processes in preterm children, we adopted a cross-population and multi-modal approach comparing eye movements in reading tasks among three groups: children with preterm birth, children with a diagnosis of dyslexia, and children with typical development. The study involved 78 participants (10.5 years). Eye movements (number and duration of fixations, amplitude and number of saccades, number of regressions) were recorded during the silent reading of two texts; cognitive and reading standardized tasks were also administered. Children with dyslexia had more fixations and more frequent and smaller saccades compared to the preterm group and children with typical development. They also showed more regressions compared to the control group. Preterm children showed shorter fixations compared to the other groups. Cognitive and reading standardized tasks confirmed severe delays in reading in children with dyslexia and some weaknesses in text reading speed and comprehension in preterm children. These results are discussed with reference to candidate mechanisms that underlie reading processes in preterm children and considering possible implications for research.

## 1. Introduction

Preterm birth, defined by the World Health Organization as a birth before 37 weeks of gestational age, is a relatively frequent event ranging from 5 to 18% in the world [1,2]. Preterm birth occurs in a sensitive period for nervous system development and exposes the newborn to not optimal environmental and socio-relational experiences for development, increasing risks for poorer long-term neurodevelopmental outcomes [3,4]. For these reasons, interdisciplinary follow-up is necessary for the preterm population, suggesting the right to continuity of post-hospital care as indicated by the European guidelines [4]. Among neurodevelopmental outcomes, preterm birth can affect academic achievements at school age, as suggested by several reviews and meta-analyses [3,5,6,7]. Concerning reading, delays are not severe: preterm children scored 0.48 *SD* lower than full-term peers on reading [5] and 0.57 on text comprehension [8].

Moreover, in orthographically regular languages such as Italian, delays in reading were persistent in speed but not in accuracy, as well as in text reading compared to single words or non-words [9]. Although findings in the literature are concordant with the presence of delays in reading speed among preterm children, the profile of very preterm children and the underlying mechanisms of these delays have yet to be deeply investigated. In the present study, two recent lines of research have been implemented to understand the profile of very preterm children in reading: cross-population studies and multi-modal approaches, specifically through the analysis of eye movements in reading tasks.

First, cross-population studies allow an understanding of the profile of preterm children beyond unique typically developing comparisons, as already suggested for other populations with atypical development, by identifying similarities and differences across disorders [10]. To our knowledge, only two studies have compared reading skills among preterm children, full-term children with typical development, and children diagnosed with dyslexia or learning disorders. The first study [9] revealed that delays in very preterm children at 10 years are less widespread and severe than those of children with Specific Learning Disorders (as mean scores are within −1 *SD*). However, the delay in reading is residual and persistent, with lower text reading speed and comprehension scores compared to typically developing children. Contrasting results were found in a second study [11], as 8–11-year-old preterm children did not show delays in reading and spelling compared to typically developing readers, whereas the difference between preterm children and children with dyslexia was confirmed. The comparison between preterm children and children with dyslexia is particularly enriching as dyslexia involves specific difficulties in reading or spelling that are not primarily due to intellectual disability or global developmental delay, neurological, motor, or sensory disorders, or a lack of opportunity for learning/inadequate instruction [12]. The etiology of dyslexia is currently interpreted within a multiple deficit model [13,14], which states that multiple predictors (e.g., phonology, attentional and visuo-perceptual processes, speed of processing) contribute probabilistically to it. Disfluency (i.e., poor reading speed and/or accuracy) is a strong behavioral marker of dyslexia, although its determinants might vary depending on the different underlying functional profiles. Although dimensional models are suggested to better frame reading impairments [15], there is evidence that decoding and spelling skills do not load on general cognitive functioning [16]; therefore, they maintain some specificity. Comparing preterm children with those with dyslexia would allow us to understand better whether reading weaknesses in the two groups share similar processes and whether reading impairments in preterm children can be considered a behavioral marker or a secondary symptom resulting from domain-general cognitive processes.

The second line of research is to understand the mechanisms affecting academic achievement in preterm children by integrating multi-modal approaches. Besides standardized reading and comprehension tasks, eye movements are helpful because they provide real-time information about the reading process. Further, they are not dependent on verbal responses, which might constitute a bias in assessment for populations, such as preterm children, having weaknesses in oral/verbal skills [3]. During reading, control of eye movements is needed to coordinate both eyes (binocular coordination) in the correct direction of reading horizontally (from left to right in western writing systems) and vertically to start a new line. Eye movement patterns include a series of fixations (i.e., the maintenance of the gaze on a definite location to extract information) and saccades (i.e., shifts of the gaze between fixations that direct the eyes onto the next target to be fixated) (see review in [17]). Important findings show that the amount of information extracted during a single eye fixation during reading is tightly linked to reading ability development [18]. In reading research, regressions (backward saccades) represent another critical parameter in the way they represent movements from right to left which usually indicates the need to reread the previous words [19].

Concerning preterm children, a fascinating insight comes from the study of [20], who compared, for the first time, analysis of reading and eye tracker in preterm children (7.8 years old) with groups of full-term children matched for chronological age (first group) or reading age (second group). Results suggested no differences in the oculomotor pattern between preterm and full-term reading age-matched children. In contrast, full-term chronological age-matched children showed shorter fixations, less frequent saccades, and larger prosaccades amplitude compared to the other groups, suggesting that the development of the brain areas is immature in the preterm population for their chronological age [20]. Some studies on typical populations indicate that reflexive movements, such as ocular following, are not age-related [21], but there are no studies on preterm populations at this regard. In the math domain, using eye-tracking, very preterm children at 10 years were slower in non-symbolic magnitude comparison with an atypical gaze in exploration [22]. These latter studies suggest the relevance of introducing the eye-tracking methodology to understand online processing when performing a specific task [23].

Whereas only one study has explored eye movements during a reading task in the preterm population, several studies have reported fascinating results in children with dyslexia, both considering reading and non-reading tasks [17,24,25,26]. As evidenced in the review by [17], children with dyslexia tend to perform more frequent saccades of smaller amplitude during a reading task, associated with a high number of fixations of longer duration. Furthermore, a high number of backward saccades have been found to re-fixate the word. However, there are some contrasting results in the literature. For example, some studies [27,28] found a similar rate of regressions in children with dyslexia and children with typical development in reading texts with a regular orthography. These controversial results could depend on the heterogeneity of the manifestations of dyslexia and cognitive correlates, or they might be connected to the different types of materials or conditions (simple vs. difficult texts) used in the experiments [29]. Concerning this latter issue, most eye movement studies focus on silent reading characteristics [30], the modality also adopted in the present study.

### The Present Study

Starting from these new lines of research, the present study combined, for the first time to our knowledge, a cross-population study with a multi-modal approach, using eye-tracking in a reading task. Indeed, two studies have compared preterm children, children with dyslexia, and typically developing children in reading tasks [9,11] without analyzing eye movements. In addition, only one study investigated eye movements in the preterm population compared to control groups without including the comparison with children with dyslexia [20]. The main aim was to compare preterm children at the end of primary school with typically developing children and children with a diagnosis of dyslexia during silent reading of two short texts, analyzing their speed and their attentional indexes collected with eye-tracker (number of fixations, duration of fixations, number of saccades, amplitude of saccades, number of regressions). In addition, we also compared the three groups on cognitive skills and standardized reading tasks to delineate group differences better.

We hypothesized that preterm children should show a different eye movement pattern than children with dyslexia, with less severe atypical patterns. More specifically, we expect to find shorter and more numerous saccades associated with longer and more frequent fixations in dyslexia compared to both preterm and control children. However, given the weaknesses reported in reading speed and comprehension in preterm children, we expect to find some atypical patterns in this group compared to the control peers. Given the paucity of studies in this field in preterm children, this is an exploratory aim.

## 2. Materials and Methods

### 2.1. Participants

The total sample of the present study comprises 78 children in the fourth and fifth grades of primary school, divided into three different groups, namely very preterm children (*n* = 18; mean age: 125.7 months, *SD* = 8.03; 8 females); children with a diagnosis of dyslexia (*n* = 18; mean age: 125.7 months, *SD* = 5.53; 8 females); and children with typical development, defined as a control group (*n* = 42; mean age: 125.6 months, *SD* = 4.59; 25 females). Children in the three groups were balanced for age and gender. Common inclusion criteria were having an intelligence quotient higher than 75 and being Italian monolingual.

The preterm group was part of a cohort of preterm children born in 2003 and 2004 at the Neonatology Unit of the University Hospital of Bologna, and they all had, at birth, a gestational age < 32 weeks, and no major cerebral damage nor congenital malformations, nor an indication of visual or hearing impairment.

Children with dyslexia were recruited at the LADA laboratory, University of Bologna. Only children with a reading and/or spelling disorder diagnosis were included in the present study. The diagnosis was based on clinical evaluation and respected the Italian criteria [31], that is, having a score of less than −2 *SD* in at least 2 of 6 parameters in tasks of word, nonword, and text reading (speed and accuracy), or spelling (words, nonwords, sentences).

Children with typical development were recruited from several primary schools in Bologna and had no congenital malformations or visual/hearing impairments.

Group social background characteristics were comparable: area of residence; educational exposure (only public schools were involved); linguistic and cultural context (all children had both Italian parents).

### 2.2. Materials

*Intellectual functioning:* The Italian version of the Kaufman Brief Intelligence Test, Second Edition [32] (K-BIT-2) was administrated. The KBIT-2 allows the calculation of three indexes, namely a Verbal score (V-IQ), a Non-verbal score (NV-IQ), and a Composite score (C-IQ).

*Speed of processing*: A task to measure simple reaction times [33] was administered. Children were required to press the keyboard’s space bar as fast as possible whenever a ‘blue star’ (measuring 8 cm × 8 cm) appeared on a white screen. The target stimulus was presented on the screen for a maximum of one second and disappeared after the response was made. The following stimuli appeared at 1-second intervals after the preceding stimulus had disappeared. Fifteen practice trials were completed, followed by 40 test trials. Mean RTs were recorded.

*Decoding and reading comprehension tasks*: To assess decoding skills, children were asked to read aloud a list of words (112 words, DDE-2, [34]), non-words (48 non-words, DDE-2, [34]), and a text (MT-2, [35]). Accuracy (number of errors) and speed (number of syllables/second) were computed for all tasks. For reading comprehension, children were asked to read a text alone and then answer 14 multiple-choice reading comprehension questions (MT-2, [35]). The number of correct responses was scored.

*Eye movements in reading tasks*: Children read silently, in sequence, two informative texts about animal life, of 64 words each, aligned on 8 rows (130 syllables for text 1 and 126 for text 2). Each text was displayed all at once on a single computer screen. After each text, children were asked to respond to a very simple multiple-choice comprehension question to ascertain that they understood the main concept of the text. All children answered correctly and therefore were kept in the study. According to Italian practice and in line with the scoring of standardized reading tasks, we coded the reading speed in terms of syllables per second (syll/sec). Before the experiment, a calibration step of the eye tracker was performed according to a 9-point grid extended to the computer screen. During the reading tasks, eye movements were recorded with a corneal reflection eye tracking system based on a remote pan/tilt infrared camera (Applied Science Laboratory Model 504). Eye position was sampled and stored at a rate of 120 Hz, with a spatial resolution of 0.25° visual/angle. Participants sat 56 cm from a 30.5 cm × 23 cm monitor screen. The area of interest (AOI) was defined within 0.5 cm around the text. We considered eye-movement data that fell within the AOI, and the interest period extended from the onset of the stimuli until the child pressed the spacebar. The following variables were then examined in the analysis:

- Number and duration of fixations: a drift in the viewer’s gaze by less than 1° within 0.1 s identified a fixation; only the fixations that lasted for at least 0.1 s were considered. The total number and the mean duration (in seconds) of fixations were calculated for each participant;
- Number and amplitude of saccades: saccades correspond to gaze shifts between fixations and are computed as the number of horizontal movements in the text from left to right, e.g., [36]; the amplitude is referred to the degrees of visual angle covered by the saccade. Saccades higher than 10° were excluded.
- Number of regressions: regressions are referred to saccades from right to left in the text, denoting a look back at the text. The number of regressions was computed.

### 2.3. Procedure

Trained psychologists assessed all children in a quiet room at the Department of Psychology, University of Bologna. The study protocol met the ethical guidelines for the protection of human participants, including adherence to the legal requirements of the country, and received formal approval from the and approved by the Independent Ethics Committee of the University Hospital of Bologna, Sant’Orsola Malpighi (prot. n. 76/2013/U/Sper, 10 January 2014). The parents of the children provided their informed written consent for their child’s participation in the study, data analysis, and for anonymous data publication.

### 2.4. Data Analysis

Group differences were analyzed with a series of univariate and multivariate analyses of variance (ANOVAs and MANOVAs), with the Group (preterm children, children with dyslexia, control group) as a between-subject factor. Concerning standardized measures, dependent variables were total IQ, verbal IQ and non-verbal IQ (ANOVAs), speed of processing (ANOVA), z-scores in reading accuracy and speed (two separated MANOVAs) for word, non-word, and text reading, and reading comprehension (ANOVA). Concerning measures of eye movements, we carried out six repeated measures ANOVAs with Text (text 1 vs. text 2) as the within-subject factor and Group as the between-subject factor. The dependent variables were: syll/sec in silent reading of texts; number and duration of fixations; number and amplitude of saccades; number of regressions. Partial eta-squares (η^2^) were reported as a measure of effect size. Tukey post hoc comparisons were also reported. Considering the exploratory nature of these analyses, multiple test adjustment was not performed [37].

## 3. Results

### 3.1. Standardized Tasks

Descriptive analyses of the variables derived from the cognitive and reading standardized tasks are reported in Table 1.

Regarding intellectual functioning, the three groups performed similarly in verbal, non-verbal, and composite IQ, with no Group main effect, F(2, 77) = 0.438–0.815, *p* = 0.419–0.647, η^2^ = 0.012–0.023. In addition, for speed of processing, the effect of Group was non-significant, F(2, 77) = 2.829, *p* = 0.065, η^2^ = 0.070.

By contrast, a significant effect of Group was found for reading accuracy, F(6, 148) = 12.165, *p* < 0.001, η^2^ = 0.330, with the univariate analysis showing significant differences among groups for all the three stimuli considered (words, non-words, text; F(2, 77) = 30.345–68.485, all *p*s < 0.001, η^2^ = 0.447–0.646). Tukey’s post hoc revealed the same pattern of results for the three variables, with children with dyslexia showing lower (*p* < 0.001) reading accuracy compared to both preterm and control children, which had similar performances. As for reading speed, a significant effect of Group was found, F(6, 148) = 10.084, *p* < 0.001, η^2^ = 0.290. In addition, in this case, univariate analysis revealed significant differences among groups for all the three variables considered, F(2, 77) = 15.896–40.112, all *p*s < 0.001, η^2^ = 0.298–0.517, with the dyslexic group showing slower (*p* < 0.001) word and non-word reading compared to preterm and control groups, which had comparable performances. In text reading, the dyslexic group had lower scores compared to preterm and control children (*p* < 0.001), but the preterm group had lower scores compared to control children (*p* = 0.029). Finally, also the ANOVA run on the reading comprehension score revealed a significant effect of Group, F(2, 77) = 10.050, *p* < 0.001, η^2^ = 0.211, with the dyslexic (*p* < 0.001) and preterm (*p* = 0.007) groups showing lower scores compared to the typically developing group, and similar scores between them.

### 3.2. Eye-Movement Variables

The descriptive data of the variables derived from the experimental task are reported in Table 2.

The analysis of the silent reading speed revealed a significant effect of Group, F(2, 75) = 10.702, *p* < 0.001, η^2^ = 0.222, whereas the effects of Text, F(1, 75) = 0.052, *p* = 0.821, η^2^ = 0.001, and Text×Group, F(2, 75) = 1.062, *p* = 0.351, η^2^ = 0.028, were non-significant. Post hoc analysis showed children with dyslexia having a weaker performance compared to both preterm (*p* = 0.002) and control children (*p* < 0.001), which obtained similar scores.

**Figure 1:**
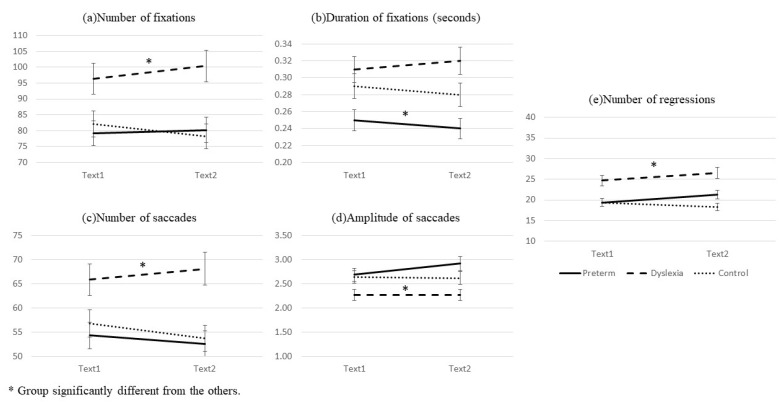
(**a**–**e**). Graphic Representation of the Eye movements in the Reading Task.

Analysis of the fixations showed a significant effect of Group for number (Figure 1a), F(2, 75) = 4.496, *p* = 0.014, η^2^ = 0.107, and duration (Figure 1b), F(2, 75) = 7.161, *p* = 0.001, η^2^ = 0.160, and non-significant effects of Text or the interaction Text×Group considering both variables, F(1/2, 75) = 0.026–1.225, *p* = 0.300–0.872, η^2^ = 0.000–0.032. In addition, post hoc analyses indicated a higher number of fixations for the dyslexic group compared to the preterm (*p* = 0.042) and control groups (*p* = 0.016), which were similar. As for fixations duration, the preterm group showed shorter durations compared to both the dyslexic (*p* < 0.001) and control (*p* = 0.046) groups, which had similar scores.

The Group effect was also significant for the number (Figure 1c), F(2, 75) = 4.592, *p* = 0.013, η^2^ = 0.109, and amplitude (Figure 1d), F(2, 75) = 6.637, *p* = 0.002, η^2^ = 0.150, of saccades, with the Text and Text×Group effects being non-significant, F(1/2, 75) = 0.273–2.449, *p* = 0.093–0.603, η^2^ = 0.004–0.061. For the number of saccades, children with dyslexia showed a higher value than the control (*p* = 0.021) and preterm (*p* = 0.025) groups, which were similar. Children with dyslexia showed lower values for saccades amplitude than the control (*p* = 0.017) and preterm (*p* = 0.002) children, which were again similar.

Considering the regressions (Figure 1d), a significant effect of Group was found F(2, 75) = 3.928, *p* = 0.024, η^2^ = 0.095. Effects of Text and Text×Group were all non-significant, F(1/2, 75) = 0.872–1.548, *p* = 0.219–0.353, η^2^ = 0.011–0.040. Post hoc analyses revealed that the dyslexic group showed more regressions than the control group (*p* = 0.018).

## 4. Discussion

The current study aimed at a cross-population comparison, namely amongst preterm children, children with dyslexia, and children with typical development, through a multi-modal approach that involved eye-movement analysis in reading tasks and standardized tests. The groups were balanced for age and gender and did not differ in verbal, non-verbal, and composite IQ, as well as in speed of processing. The absence of differences in IQ and speed of processing measures confirms previous studies on children with dyslexia [33]; and preterm children [9,22] and allows us to exclude that differences in reading tasks and eye movement patterns were due to cognitive functioning.

Considering standardized measures of reading skills, preterm children performed similarly to the control group in word and non-word reading accuracy and speed and text reading accuracy but underperformed in text reading speed. Moreover, they showed lower scores in reading comprehension. Children with dyslexia, as expected, underperformed in all reading tasks and were similar to preterm children in reading comprehension. These results align with previous findings [9], showing that the reading profile of preterm children falls within the normal range but shows weaknesses at the text level [8]. However, as reading is affected by orthographic consistency, our findings can be generalized to preterm children exposed to written languages with similar regularity in orthography. Indeed, persistent delays only in reading speed were described by previous studies in orthographically regular languages, such as Italian [9]. In contrast, delays in both reading accuracy and speed were found in preterm children exposed to opaque languages [5]. Ultimately, our results are related to the population of very preterm children characterized by a gestational age < 32 weeks of gestation. By contrast, delays are less evident or absent in preterm children with moderate risk and higher gestational age, including, for example, children born at 33 weeks of gestation [11].

Regarding the online processes that occur in reading tasks, our results on children with dyslexia generally confirmed previous findings [17,18,29,30], with more frequent fixations, a higher number of saccades with reduced amplitude, and more regressions compared to the control group. To note, previous research on transparent languages found contrasting results in regression patterns in children with dyslexia, with some finding significant differences compared to the control group [30] and others not [27,28]. Besides variations in text structure, discrepancies might be explained concerning sampling procedures. In the present study, as in [38], inclusion criteria were more severe (present study: less than −2 *SD*; [30] −1.65 *SD* in reading tasks) than those used in other studies; therefore, the higher number of regressions might be related to the severity of the impairment.

Our study is explorative concerning the eye-movement pattern of preterm children in silent reading as only one study has been carried out on this issue [20]. Our results revealed a pattern similar to that of children with typical development except for the duration of fixations. Indeed, preterm children had shorter fixations, both with respect to control children and, particularly, to the dyslexic group. Our results differed from [20], as they found an opposite trend with a longer duration of fixations in preterm and full-term reading age-matched children compared to the chronological age-matched children group. In addition, the two first groups also showed a higher number of pro- and backward saccades and a higher amplitude of pro-saccades compared to the latter group, suggesting an immature development of brain areas in preterm children compared to their chronological age peers. Methodological differences can partially explain these contrasting results between the two studies. First, we included very preterm children (gestational age < 32 weeks of gestation), whereas in the other study [20], only extremely preterm children were included with a range of gestational ages between 24 and 28 weeks. The second difference concerned the age of the participants. Indeed, our children were 2.5 years older than those in the other study [20]. Taking into account these differences, further studies are needed to understand if these different patterns described in the preterm population can be due to a different development of reading brain areas in function of neonatal immaturity grade and age, with more evident atypical oculomotor patterns in extremely preterm children [20] at a younger age and more shading difficulties in very preterm children at the end of primary school. Previous studies seem to suggest different atypical oculomotor patterns in the preterm population in relation to neonatal immaturity and age. Indeed, Bucci and colleagues [39] described in extremely preterm children at 8 years immature brain structures controlling eye movements, particularly the parietal and frontal cortexes, during other tasks not involving reading. By contrast, in very preterm children at the end of primary school, shorter fixations have been described using eye-tracker in other domains. Indeed, very preterm children showed more and shorter fixations in non-symbolic magnitude comparison tasks [22]. These results seem to be in line with previous studies on infant preterm children that showed that before 12 months of age, preterm infants tended to spend less time focusing on the referential object/target [40,41,42], interpreted as poor attentional control and/or inhibition deficits.

Concerning the comparison between preterm children and children with dyslexia, it is to note that there was a wide discrepancy in fixation length, as children with dyslexia had significantly longer fixations than preterm children. This pattern suggests that the online reading process of the two groups differ at a qualitative level: children with dyslexia need more time for each fixation, possibly because of limited connectivity and the need to match the correct sound to the letter [27,43], whereas preterm children show sorter fixations, possibly for poor attentional control.

To sum up, this study analyzed eye-movement patterns during reading tasks among three different populations and provided evidence that preterm children and children with dyslexia behaved differently, despite both groups being slower in text reading compared to typically developing children at a behavioral level, as suggested by standardized tasks. This is the first study of its kind to examine eye-movement patterns among these three populations. The eye-movement pattern of preterm children was much more similar to that of the typically developing population. Still, their distinctive trait, particularly in reference to children with dyslexia, was that of having shorter fixations.

Some limitations of the current study need to be considered for generalizing our findings. First, the sample size was relatively small, and we included very preterm children. Therefore, a larger sample would prove helpful to investigate the role of neonatal immaturity on eye movements during reading skills. Secondly, the reading task was coded as a whole, and we did not examine variations according to linguistic variables (i.e., word frequency and complexity). The analysis of gaze behavior in more specific reading tasks would be very useful for understanding whether this atypical pattern of exploration is consistent across different types of stimuli. Third, in the present study, only voluntary eye movements were considered. Previous studies did not find an age-related difference in ocular following responses (OFRs) [21], suggesting that reflexive, non-voluntary movements might offer new insights into understanding typical and atypical patterns in preterm children and children with dyslexia.

## 5. Conclusions

The present study offered a new perspective on the analysis of reading skills in preterm children, suggesting the need to investigate further the online processing of reading skills in this population and integrate other measures to shed light on brain development. A strength of the present paper is that of being the first cross-population study on preterm children, children with dyslexia, and typically developing children adopting an analysis of eye movements in reading tasks. Results highlighted some trends that need future investigations, also considering the above-discussed limitations of the study, particularly the need for a more fine-grained analysis of eye movements adopting research paradigms on different types of reading stimuli. A fascinating insight comes from the fMRI study [44], which tested reading skills in very preterm children at 8 years. They found that, whereas in the full-term sample, the individual variation of reading abilities was mainly explained by axonal features, in the preterm sample, which performed slightly lower in reading tasks, reading performance was mainly explained by myelin content. As also highlighted by [17], adopting multi-modal approaches that combine eye movements and ERP or fMRI techniques would allow a better understanding of real-time processes that can offer new perspectives on cognitive, neural, and behavioral markers of atypical development.

## Figures and Tables

**Table 1:** Means and Standard Deviations for the Cognitive and Reading Variables.

	Variable	Preterm	Dyslexia	Control
Cognitive variables	Verbal IQ	109.56 (13.69)	114.94 (12.60)	116.21 (13.04)
	Non-verbal IQ	99.67 (14.66)	100.61 (12.23)	102.64 (10.92)
	Composite IQ	102.39 (12.16)	106.72 (13.25)	106.14 (9.61)
	Speed of processing	247.71 (78.21)	279.83 (75.80)	238.80 (44.61)
Reading variables *	Word reading—accuracy	−0.07 (1.14)	−1.94 (1.89)	0.51 (0.54)
	Non-word reading—accuracy	−0.07 (1.47)	−1.94 (1.21)	0.34 (0.59)
	Text reading—accuracy	−0.13 (1.14)	−2.84 (1.49)	0.34 (0.53)
	Word reading—speed	0.48 (0.91)	−1.02 (1.01)	0.90 (1.10)
	Non-word reading—speed	0.33 (0.84)	−0.99 (0.63)	0.53 (1.13)
	Text reading—speed	0.17 (0.61)	−0.99 (0.54)	0.65 (0.70)
	Reading comprehension	−0.58 (1.43)	−0.85 (1.02)	0.34 (0.87)

* For the reading variables, z-scores obtained based on the tests’ norms are reported.

**Table 2:** Means and Standard Deviations for the Eye-movement Variables.

Variable		Preterm	Dyslexia	Control
Silent reading speed (syll/sec)	Text 1	4.79 (1.21)	3.07 (1.26)	4.73 (1.41)
	Text 2	4.60 (1.14)	3.26 (1.73)	4.80 (1.20)
Number of fixations	Text 1	79.17 (23.09)	96.39 (34.00)	82.12 (19.95)
	Text 2	80.17 (24.84)	100.39 (37.43)	78.23 (26.16)
Duration of fixations (seconds)	Text 1	0.25 (0.07)	0.31 (0.07)	0.29 (0.04)
	Text 2	0.24 (0.06)	0.32 (0.06)	0.28 (0.05)
Number of saccades	Text 1	54.28 (16.04)	65.83 (22.33)	56.76 (13.79)
	Text 2	52.56 (17.71)	68.11 (23.17)	53.69 (12.25)
Amplitude of saccades (degrees of visual angle)	Text 1	2.69 (0.43)	2.27 (0.43)	2.64 (0.49)
	Text 2	2.92 (0.68)	2.27 (0.38)	2.62 (0.51)
Number of regressions	Text 1	19.39 (8.74)	24.67 (13.41)	19.40 (7.49)
	Text 2	21.22 (9.96)	26.56 (14.54)	18.26 (5.67)

## Data Availability

Data will be made available from authors upon reasonable request.
